# Pentraxins: Structure, Function, and Role in Inflammation

**DOI:** 10.1155/2013/379040

**Published:** 2013-09-14

**Authors:** Terry W. Du Clos

**Affiliations:** ^1^The Department of Veterans Affairs Medical Center, Research Service 151, 1501 San Pedro SE, Albuquerque, NM 87108, USA; ^2^Department of Internal Medicine, The University of New Mexico School of Medicine, Albuquerque, NM 87108, USA

## Abstract

The pentraxins are an ancient family of proteins with a unique architecture found as far back in evolution as the Horseshoe crab. In humans the two members of this family are C-reactive protein and serum amyloid P. Pentraxins are defined by their sequence homology, their pentameric structure and their calcium-dependent binding to their ligands. Pentraxins function as soluble pattern recognition molecules and one of the earliest and most important roles for these proteins is host defense primarily against pathogenic bacteria. They function as opsonins for pathogens through activation of the complement pathway and through binding to Fc gamma receptors. Pentraxins also recognize membrane phospholipids and nuclear components exposed on or released by damaged cells. CRP has a specific interaction with small nuclear ribonucleoproteins whereas SAP is a major recognition molecule for DNA, two nuclear autoantigens. Studies in autoimmune and inflammatory disease models suggest that pentraxins interact with macrophage Fc receptors to regulate the inflammatory response. Because CRP is a strong acute phase reactant it is widely used as a marker of inflammation and infection.

## 1. Introduction

In this review, I focus on the two major, classical pentraxins: C-reactive protein (CRP) and serum amyloid P component (SAP). The pentraxins are serum proteins with a relatively uncommon pentameric structure. They function as pattern recognition molecules recognizing foreign antigens and altered self-antigens and tag these molecules for activation of the innate immune system. This property is characteristic of innate recognition molecules that preceded the development of the immunoglobulins. Pentraxins also interact with the complement system and Fc receptors to activate immune responses. It is likely that the interaction of pentraxins with the receptors for the Fc region of immunoglobulins preceded the development of immunoglobulins. 

## 2. History

The pentraxins appeared very early in evolution with several different forms present in the horseshoe crab, which has been referred to as a living fossil having persisted 250–300 million years [1]. Despite this long lineage, our understanding of the function of these proteins remained obscure until very recently. The discovery of CRP in man was achieved serendipitously in the blood of a patient with severe *Streptococcus pneumoniae* pneumonia. The protein appeared in the blood when the patient was systemically ill and was not detectable before the infection or after the infection had been eradicated [2]. These clinical investigators at the Rockefeller University went on to characterize this protein biochemically. The protein was present in very high concentration in acute phase sera and it would induce precipitation of pneumococcal cell wall extracts but only in the presence of calcium. 

## 3. Pentraxin Structure

The molecular mass of CRP and SAP is 115,135 daltons and 127,310 daltons, respectively. Both proteins are composed of five tightly arranged subunits (protomers) in planar symmetry. Using electron microscopy, it was determined that the molecule appeared as a doughnut-shaped ring [3]. Although it was a long held belief that CRP was composed of a single pentamer whereas SAP existed as a decamer, it was later determined that SAP, like CRP, circulates in blood as a single pentamer [4]. The pentameric structure of CRP imparts a high degree of stability to the molecule and resistance to enzymatic attack [5]. SAP shares many structural and biological characteristics with CRP. They are both cyclic pentamers that react with ligands in a calcium-dependent fashion. They share 51% amino acid identity and very similar structures [6]. Unlike CRP, SAP is glycosylated and the carbohydrate moiety has been defined [7]. See Table 1 for a comparison of CRP and SAP.

**Figure 1:**
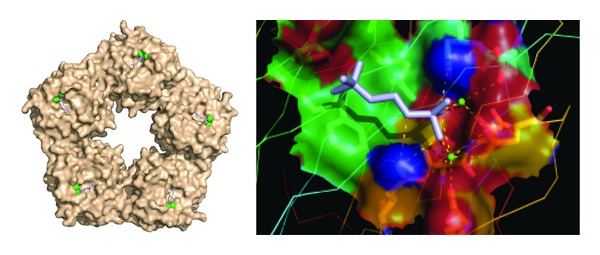
Surface view of the ligand binding (B) face of C-reactive protein.  Each protomer contains a binding site, which is shown occupied by 2 calcium (green) and 1 PC molecule (blue). On the magnified view on the right the major interactions with bound calcium ions and specific amino acids can be seen more clearly. The structure is taken from structure file PDB ID: 1B09 from the NCBI.

**Figure 2:**
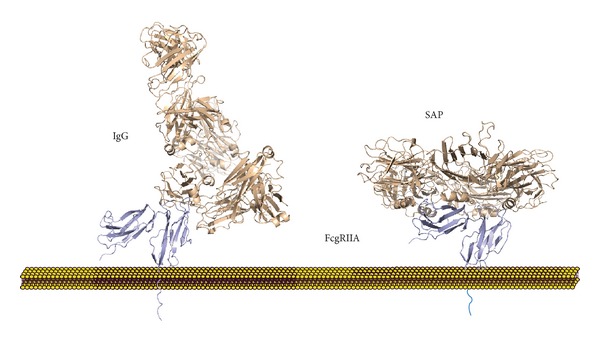
Comparison of the binding of SAP and IgG to the Fc*γ*RIIA molecule. The structure of the IgG-Fc*γ*RIIA is shown on the left and is based on the NCBI entry 3RY6. The structure of the SAP-Fc*γ*RIIA complex taken from the NCBI entry 3D5O is shown on the right [14]. The FcR interaction with SAP engages the ridge helices of two nonadjacent protomers, resulting in a one-to-one stoichiometry.

The first crystallographic structure of the pentraxin family was solved for SAP, which revealed the five-fold symmetry of the molecule and the calcium-dependent binding site for the 4,6-cyclic pyruvate acetal of *β*-D galactose and phosphoethanolamine [8]. The structure of CRP was definitively determined when the first crystallographic model was reported [9]. Each pentraxin protomer consists of a conserved *β*-sandwich fold with two opposing *β*-sheets. The binding site for phosphocholine (PC) was proposed to be a hydrophobic pocket on one face of the protomer, and similarities and differences between the CRP and SAP binding sites were observed. Shortly thereafter a crystallographic solution of the interaction between CRP and the PC ligand was produced [10].  Figure 1 shows the ligand binding sites on CRP with two calcium ions forming part of the site. On the other face of the protomer was found a three-turn alpha helix, termed the ridge helix, and a deep groove of uncertain function. This face of the CRP pentamer was shown by mutational analysis to contain a single C1q binding site [11, 12] and by mutational analysis and cocrystallization to contain a single Fc receptor binding site [13, 14] (Figure 2).

Another group of related proteins was described more recently and is known as the long pentraxins. The long pentraxins share a strong homology with the pentraxins in the C-terminal region but have a long N-terminal domain that is unrelated to the so-called short pentraxins or other known proteins. These “long pentraxins” are not as well structurally characterized yet and their functions less defined. Unlike the classical pentraxins, the long pentraxins are produced locally in response to inflammatory stimuli like TNF-*α*. The long pentraxins include guinea pig apexin, neural pentraxin I (NPTXI) and II (NPTXII), and long pentraxin 3 (PTX3). PTX3, the best studied of these, activates complement, binds to Fc*γ*RIII [14], protects from some fungal infections [15, 16], and may play a role in wound healing. The long pentraxins have been reviewed recently [17, 18]. 

## 4. Ligands Recognized

Much of the early work on CRP biology focused on its interaction with ligands expressed on bacteria and damaged tissue. CRP was initially identified and named for its interaction with the C-polysaccharide, a major component of the cell wall of *S. pneumoniae* [19]. CRP binding to the C-polysaccharide was shown very early on to occur through PC moieties, which are found on the cell wall teichoic acid and lipoteichoic acid [20]. Also see Figure 1. The binding to PC was calcium dependent. PC is expressed on a variety of pathogenic organisms to which CRP has been shown to bind. PC is also the polar head group of phosphatidylcholine, a component of the mammalian cell membrane. This PC head group of phosphatidylcholine is not exposed on normal healthy cells. However, damage to cell membranes by enzymatic action or complement attack leads to extensive binding of CRP to the damaged membrane [21, 22]. This was first demonstrated *in vivo* by injecting typhoid vaccine into rabbit muscle and examining CRP deposition [23]. Subsequently, similar results were obtained when coronary artery ligation was used to induce myocardial infarction [24]. Thus CRP can target dead and damaged cells for processing by the innate immune system. CRP also binds to PC exposed on oxidized LDL, which may account for its presence in atherosclerotic lesions [25, 26].

The damaged cell can present and/or release various nuclear antigens that can stimulate the immune system and some of these are the targets of autoantibodies in connective tissue diseases. The most notable of these is systemic lupus erythematosus (SLE) in which patients develop high-titered antibodies to native DNA and ribonucleoprotein complexes [27]. CRP and SAP bind to these nuclear antigens and affect their clearance and antigenic processing. In cells CRP binds primarily to the small nuclear ribonucleoproteins (snRNPs) [28] and SAP binds to chromatin and native DNA [29, 30]. 

CRP binding to polycations has been reported and characterized [31–34]. CRP binding to polycations differs from binding to prototypic ligands such as PC in that it is inhibited by calcium and not by PC. No physiological role for CRP binding to polycations has been described. However, polyvalent binding to either type of ligand leads to complement activation through C1q [35, 36].

SAP has similar ligand binding sites on the B face of the molecule, but whereas it binds well to PE, it fails to bind to PC due to differences in the hydrophobic pocket [6, 8, 37]. SAP binds to DNA as well although the affinity is much stronger for human SAP than for mouse SAP [38]. This difference may complicate the study of the role of pentraxins in mouse models of SLE. SAP binds to other polyanions, including heparin, to carbohydrates on bacteria including *Streptococcus pyogenes* and *Neisseria meningitidis*, and to lipopolysaccharide (LPS) [39, 40]. The SAP ligand in agarose was identified as the 4,6-cyclic pyruvate acetal of *β*-D-galactose [41]. SAP binds to and is a constituent of all types of amyloid fibrils [42]. This ability to bind to amyloid is the basis of an assay to localize amyloid deposits in patients with amyloidosis [43].

## 5. Pentraxins and Complement

One of the first breakthroughs in pentraxin biology was the finding that CRP could activate the classical cascade of complement [36, 44]. This finding suggested an important biological function for CRP as the complement system has a broad range of activities in biological defense and regulation of inflammation [45]. CRP activates the classical cascade of complement through direct binding of C1q, the first component of the classical pathway. Each CRP pentamer has a single binding site for C1q and a minimum of two CRP molecules are required for C1 activation, similar to IgG [46]. No crystallographic solution of the CRP-C1q interaction has been produced to date. It was originally reported that CRP interacted with the collagen-like stalk of the A chain of C1q [47, 48]. However, more recently CRP interactions with C1q globular head groups were reported [49]. A molecular model has been presented in which one globular head group of C1q interacts through the central pore of CRP on the A face of the pentamer [50]. This model is based on site-directed mutagenesis studies of the CRP binding site for C1q [11, 12]. SAP either chemically cross-linked or bound to polyvalent ligands also binds C1q and activates the classical complement pathway [51, 52].

**Figure 3:**
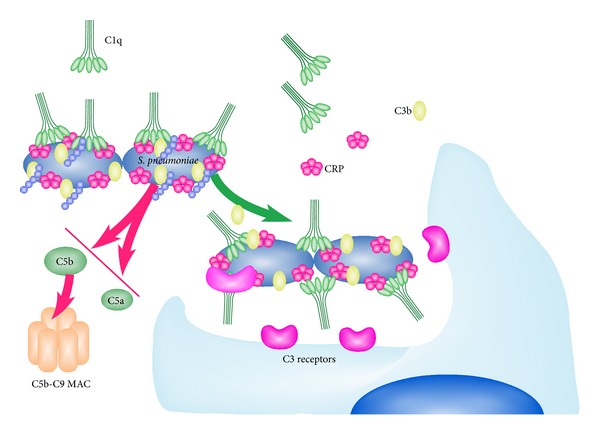
Activation of the complement cascade by CRP complexes. CRP interacts with bacteria that express repeating ligands like PC on the Gram-positive pathogen *S. pneumoniae*. A single CRP pentamer interacts with one globular head group of a C1q molecule. Interaction of C1q with multiple CRP molecules leads to C1 activation, C4 and C2 cleavage, and the formation of a C3 convertase. The cleavage of C3 in turn forms a C5 convertase. This step is limited by CRP recruitment of the inhibitory protein fH. Thus the cleavage of C5 resulting in C5a generation and formation of the MAC is blocked.

Complement activation by CRP is, at first glance, very similar to complement activation by IgM or IgG immune complexes. However, a more detailed comparison reveals that CRP activation does not efficiently proceed to generation of the membrane attack complex, whereas antibody activation does [53]. See Figure 3. CRP activates early steps in the classical pathway, with nearly complete consumption of C1, C4, and C2 and partial consumption of C3, but produces only minimal activation of C5–C9 and no cell lysis. Since C5a and C5b-9 are the strongest inflammatory mediators produced during complement activation, this restricted complement activation is likely to favor opsonization without a strong inflammatory response. Consistent with this hypothesis, CRP was shown to prevent lysis of apoptotic cells by complement, promoting opsonization and increasing anti-inflammatory cytokines [54].

Additional studies established that the characteristic early classical pathway activation by CRP is due to inhibition of the alternative pathway convertase, which provides an essential amplification loop for both the classical and lectin pathways [55, 56]. This feedback loop is especially important for forming the C5 convertase, generating inflammatory mediators, C5a and C5b-9, and contributing to complement-mediated pathology [57]. The inhibitory effect of CRP on alternative pathway activation required the complement regulatory protein, factor H (fH), and CRP was shown to recruit fH to the activating surface [55]. CRP binding to the related regulatory protein, C4b binding protein has also been reported [58].

More recent investigations have identified at least two binding sites on fH for CRP [59–61]. One of these has received particular attention because it includes the polymorphic residue (Y402H) in short consensus repeat 7 of fH that is genetically linked to the risk of developing age-related macular degeneration [62–65]. Several groups have reported that CRP binds with lower affinity to the fH variant (H402) that is associated with the disease [66, 67]. These findings suggest that although elevated serum CRP levels are associated with the chronic inflammatory process in age-related macular degeneration [68], the ability of CRP to bind fH may have a protective role in this disease. 

An important role for CRP activation of complement was shown in *S. pneumoniae* infection models where CRP activation of complement contributed substantially to protection from lethal infection and clearance of bacteria [69–72]. There is also some evidence for complement activation by CRP in acute injury, such as myocardial infarction [73]. Complement activation by CRP was reported to contribute to ischemia-reperfusion injury in a rat model of myocardial infarction although the findings are difficult to interpret because in this model endogenous rat CRP played no role and infusion of human CRP was required to activate rat complement [74]. 

## 6. Receptor Binding

Another mechanism by which CRP interacts with the innate immune system is through its interaction with Fc*γ* receptors (Fc*γ*Rs). Fc*γ*Rs are a family of membrane receptors found on myeloid cells, B lymphocytes, NK cells, and platelets. In man, they exist in three major classes with multiple subtypes [75] (Table 2). For many years it was thought that CRP could interact with cells of the immune system by binding to a specific “CRP receptor” [76]. A great deal of effort was spent searching for the specific CRP receptor. It was first proposed that CRP might interact with an Fc receptor or a receptor that was somehow associated with the Fc receptors. It was later concluded by these investigators that CRP did not bind to Fc receptors but to its own specific receptor [76]. However, numerous attempts to clone this receptor by others and by us failed to produce meaningful results. Reexamination of the cells to which CRP bound and inhibition studies suggested to us that Fc*γ*R could indeed be the direct receptors for CRP. Final identification of the receptors for CRP on leukocytes was made possible through the cloning and expression of the Fc receptors on transfected cell membranes. Using transfected cell lines we first determined that CRP was capable of binding to cells through the high affinity receptor for IgG, Fc*γ*RI [77]. Other laboratories confirmed the interaction of CRP with Fc*γ*RI [78, 79]. It was further shown that the interaction of CRP with Fc*γ*RI on transfected cells was markedly increased by the cotransfection of the cells with *γ*-chain [80]. Thus surface plasmon resonance (SPR) studies of CRP interaction with Fc*γ*RI in the absence of the *γ*-chain may underestimate the true affinity for Fc*γ*RI.

Although it was shown that CRP did bind to Fc*γ*RI, it could not explain binding to cells in which Fc*γ*RI is not expressed, for example, K562 cells and platelets. Thus, a second receptor for CRP on leukocytes was sought. Ongoing studies of Fc receptor biology made it clear that the expression of Fc receptors varied among different leukocyte subsets and that different individuals could express different numbers of receptors with differing affinities. We were able to show that a large fraction of the remaining binding was due to CRP interaction with Fc*γ*RIIA, a receptor that is responsible for many of the functions induced by immune complexes [81]. The binding of CRP to Fc*γ*RIIA was also verified independently [82]. The affinity of this interaction could not be determined quantitative terms from the flow cytometry assays [81], but an equilibrium *K*_*D*_ of 3.7 ± 1 *μ*M was determined by confocal analysis of transfected cells [82]. This is in agreement with the *K*_*D*_ of 1.9 ± 0.6 *μ*M determined for CRP binding to Fc*γ*RIIA by SPR [14]. 

Fc*γ*RIIA is expressed in two forms in humans resulting from a single amino acid polymorphism at position 131, which may be either an arginine (R) or a histidine (H) [83]. This single amino acid difference results in a preferential binding of IgG2 to the H131 form of the receptor [84]. Using population studies it has been determined that this polymorphism is associated with an alteration of risk for a wide variety of human diseases including SLE, infection, myocardial infarction, and malaria [85–88]. Interestingly, CRP binds preferentially to the R131 form of Fc*γ*RIIA [14, 89, 90]. The differential binding of CRP to the R form of Fc*γ*RIIA results in much stronger responses in PMN and monocytes [89, 91]. Recently it was demonstrated that CRP stimulates neutrophil calcium signaling in an Fc*γ*RIIA allele-specific manner [92], which is consistent with previous findings [89].

One way in which investigators have sought to decipher the conflicting data concerning CRP's direct role at the cellular level is through the use of purified CRP, which may be purified from human fluids or recombinant protein production. In our experience and that of others [93] commercial recombinant CRP preparations are often contaminated with LPS and potentially other microbial products. This can lead to effects that are directly or indirectly related to toll-like receptor (TLR) activation. In addition, most of these studies are done with uncomplexed CRP. It is well known that Fc*γ*R aggregation by immune complexes is necessary for receptor activation [94]. If that were not the case, the levels of IgG in blood would constantly bind and activate cells. Thus, CRP, like IgG, is unlikely to activate receptors without crosslinking by ligands or aggregation due to storage conditions. Clearly, ligands that contain repeating determinants like PC on pathogenic bacteria would be an optimal platform for activation. Structural and isothermal titration calorimetry studies revealed a one-to-one stoichiometry between SAP or CRP and Fc*γ*R [14] (Figure 2). It has also been shown that the degree of receptor crosslinking affects the cytokine profile of the responding cells [95]. This very likely is the reason that some groups fail to demonstrate effects of CRP on cytokine or other responses using purified, uncomplexed CRP [93]. It is worth noting that CRP-mediated activation of complement also requires binding to multivalent ligands [46]. Like IgG in circulation even high concentrations of CRP do not activate cells or complement without a relevant ligand. 

The interaction of CRP with cell surface receptors was expanded when SPR studies were performed with other related receptors. CRP did not react with FcRn, neonatal Fc receptor and no interaction was seen between CRP and the IgE receptor, Fc*ε*RI. However, it was determined that CRP bound to Fc*α*RI (the IgA receptor also known as CD89) with an affinity that was comparable to its affinity for Fc*γ*R. The interaction with CD89 was functional as phagocytosis, and signaling and cytokine production was seen [96]. The *in vivo* functional consequences of this interaction await further studies.

After identification of Fc*γ*RIIA as the main receptor for CRP, signaling through this receptor was confirmed by Chi et al. [97] using HL-60 cells differentiated to a neutrophil type with DMSO. CRP induced tyrosine phosphorylation of human Fc*γ*RIIA and Syk, as well as inducing both phosphorylation and membrane localization of phospholipase C*γ*2. This signaling pattern is what would be expected for IgG mediated signaling through ITAM bearing Fc*γ*R [75]. 

Most functional studies of CRP activation of Fc*γ*R have focused on innate immune cells, monocytes, macrophages, neutrophils, and dendritic cells. These are discussed in more detail below. An interesting example of CRP activation through Fc*γ*RIIA and Fc*γ*RIIC, which are activating forms of Fc*γ*RII, on myeloma cells was reported by Yang et al. [98]. These investigators found that primary myeloma cells and stressed myeloma cell lines bound CRP through Fc*γ*RIIA and Fc*γ*RIIC. CRP activated Akt, pERK, and NF-*κ*B signaling pathways in these cells, led to increased IL-6 synthesis, and protected the myeloma cells from chemotherapy-induced apoptosis. The results were verified *in vivo* in myeloma SCID and SCID-human mouse models.

The structural basis of pentraxin-Fc*γ*R interaction was established when the crystal structure of SAP bound to Fc*γ*RIIA was solved [99] (Figure 2). This structure shows a single Fc*γ*R interacting with the ridge helices of two nonadjacent SAP protomers thus fixing the stoichiometry at one-to-one. Pentraxin and IgG binding sites for Fc*γ*R are partially overlapping and competitive binding is seen by SPR. 

## 7. CRP and SAP Synthesis

Circulating CRP is synthesized primarily by the liver at very low levels constitutively [100]. The liver-specific transcription factor, hepatic NF-1 binding to its consensus sequence, regulates cytokine-independent CRP synthesis [101]. During the acute phase response, CRP transcription responds primarily to IL-6 and this response is enhanced by IL-1*β*. IL-6 induction is mediated by the main IL-6 activated transcription factors, STAT3 [102] and C/EBP*β* [103], which bind to response elements in the CRP promoter. In clinical trials of a mAb directed against the IL6R there was a dramatic decrease in CRP levels, supporting the important role of IL-6 in acute phase CRP synthesis [104]. CRP and serum amyloid A (SAA) are the two human acute phase proteins that show the greatest dynamic range. CRP baseline serum concentrations average less than 1 *μ*g/mL, but acute phase concentrations, are commonly in the range of 10–500 *μ*g/mL. The increase in CRP levels following an acute phase stimulus is very rapid with blood levels peaking at 48 h [105]. More importantly, CRP levels also decrease rapidly after resolution of the inciting event has occurred. This makes it more useful than the widely measured erythrocyte sedimentation rate (ESR), which remains elevated long after the inflammatory state has resolved.

Human SAP is also produced in the liver at constitutive serum levels that average 33 *μ*g/mL in women and 43 *μ*g/mL in men [106]. Although SAP is not an acute phase protein in man, it is a very strong acute phase marker in the mouse where CRP is expressed at low levels (<5 *μ*g/mL) constitutively [107]. Baseline levels of mouse SAP differ considerably among strains. Mouse SAP is induced by IL-6, similar to CRP in man [107]. Both CRP and SAP activate the complement cascade. Similarly SAP also binds to Fc*γ*R and this binding results in opsonization for phagocytosis by human or mouse phagocytes [14, 108].

## 8. CRP and SAP in Mouse Models

Several approaches have been used to investigate the function of CRP *in vivo*. Most studies have used mouse models of infection, inflammation, or autoimmune disease. Since mice express low levels of endogenous CRP, human or in some cases rabbit CRP has been used in these experiments. Fortunately both human and rabbit CRPs bind to mouse Fc*γ*R and activate mouse complement [108, 109]. In short-term disease models, injection of purified human CRP is effective. However, repeated injection is not possible because of the development of anti-CRP antibodies [110]. Another approach has been the development of transgenic mice expressing human CRP or rabbit CRP with expression controlled by either the human CRP promoter as an acute phase reactant or a diet-inducible promoter [109, 111]. Recently CRP-deficient mice were established in several laboratories although these have not yet been reported on extensively [112, 113]. For the most part studies with CRP transgenic (tg) mice and passively administered CRP have produced similar results. SAP is an acute phase protein in the mouse and SAP-deficient mice have also been studied in several disease models [40, 114–116]. These results in infection, inflammation, autoimmune, and cardiovascular disease models are summarized in the following sections. 

## 9. Pentraxins and Protection from Infection

CRP binding to *S. pneumoniae* was the first indication that CRP might participate in protection from infection. Kindmark's group first showed the opsonic activity of CRP for *S. pneumoniae* and *E. coli* [117–119]. It was subsequently demonstrated that CRP could protect mice from experimental infection with *S. pneumoniae* [120] and that this effect was mediated in large part through activation of complement [121]. Protection from pneumococcal infection was also seen when human CRP was expressed from a transgene [71]. Similarly CRPtg mice were protected from infection by *Salmonella typhimurium* [122]. CRP recognizes pathogens through recognition of PC expressed on the surface of *S. pneumoniae*, *Hemophilus influenza*, and other pathogenic organisms. Mutagenesis studies have determined that the PC-binding pocket is necessary for protection in the *S. pneumoniae* model [123]. Weiser et al. determined that expression of PC on *H. influenzae* allowed for CRP binding and killing by complement [124]. Furthermore it was shown that CRP is expressed in the respiratory tract and could be found in these secretions [125]. Thus CRP may provide a barrier function, much like IgA and a direct protective effect from respiratory tract pathogens through complement activation. 

Although SAP binds preferentially to ligands containing phosphoethanolamine, it has been shown to bind to *S. pneumoniae*, which results in classical complement activation and enhanced phagocytosis [114]. Thus CRP and SAP can both participate in protection from *S. pneumoniae,* a common and often fatal infection in the young and the elderly.

SAP binds to the LPS component of the Gram-negative cell wall, and the effects of SAP deficiency on Gram-negative infection as well as LPS shock have been studied with conflicting results. One group reported increased resistance to LPS shock in SAP^−/−^ mice [126]. A second group reported that SAP^−/−^ mice were more susceptible to LPS shock and to *E. coli* 0111:B4 but more resistant to lethal infection with *Streptococcus pyogenes* or *E. coli* J5, organisms to which SAP binds [40]. Human SAP binds to and neutralizes *Shiga* toxin 2, the main mediator of sever hemorrhagic colitis and hemolytic uremic syndrome that occurs following ingestion of enterohemorrhagic *E. coli* O157:H7 [127, 128].

Recently, it has been reported that SAP is an inhibitor of influenza viral infection [129, 130]. SAP was found to inhibit viral binding to hyaluronic acid in a calcium-independent manner. These results were consistent with earlier studies of SAP and viral infection [131, 132] although the mechanisms of action were deemed to be different. 

SAP has also been found associated with invasive *Candida albicans* and amyloid associated with this fungal infection in the gut [133]. No functional consequences were examined although it was speculated that SAP might inhibit the neutrophil response.

CRP has also been shown to bind to nonbacterial pathogens. CRP was found to bind avidly to *Leishmania donovani*. Binding was specific for the lipophosphoglycan on the surface of metacyclic *L. donovani* [134]. The result of this binding was a kind of silent phagocytosis that did not induce cytokine production or protect the host from infection. 

Others have examined the interaction of CRP with malarial parasites. Early studies indicated that CRP bound to *Plasmodium falciparum* and *P. yoelii* sporozoite surface membranes and that CRP could protect rats from experimental infection with *P. yoelii* sporozoites [135]. CRP elevation has been proposed as an excellent measure of parasitemia in falciparum malaria [136].

A correlation between genetically determined levels of CRP expression and malaria infection was carried out in a Sudanese population. This study examined an upstream polymorphism in the CRP gene, −286 (C > T > A) that is known to influence CRP levels. The A form has the highest levels of baseline CRP expression. In this study, a cohort of 192 Sudanese donors followed for malaria infection for 9 years had their CRP −286 gene locus genotyped. The prevalence of the CRP alleles A, C, and T were 21%, 52%, and 27%, respectively. The number of malaria episodes experienced by each individual over the study period was used as an index for malaria susceptibility. The A-allele, unlike the C- and T-alleles or CRP genotypes, was significantly associated with an increased number of malaria episodes, *P* = 0.007 and increased parasite counts. The proportion of A-allele carriers among donors not known to have had malaria during the study period was 18%, whereas it was 43% and 63% among donors who had experienced 1–4 and ≥5 malaria episodes, respectively, over the same period (*P* = 0.002). A second study was done in Ghana on the genetic association between Fc*γ*RIIA 131R/H polymorphisms and malaria. Using a recessive model the Fc*γ*RIIA R allele, which has higher binding to CRP and lower binding to IgG, was positively associated with severe malaria, but not with cerebral malaria [137]. Together these epidemiological and genetic associations suggest that, in addition to its utility as a prognostic marker in falciparum malaria [136], CRP may play a deleterious role in the disease.

## 10. CRP and SAP and the Kidney

CRP deposition in the kidney has been demonstrated in various forms of glomerular injury. Salmon's group found CRP deposited in renal glomeruli from patients with lupus nephritis [138]. They further suggested that the R form of the Fc*γ*RIIA gene was associated with more severe renal disease. The R form of Fc*γ*RIIA is the form that binds CRP more avidly than the H form. Szalai et al. also found CRP deposited in CRPtg (NZB x NZW) F1 lupus prone mice and showed by *in situ* hybridization that it was produced locally [139]. More recently Sjöwall et al. reported colocalization of IgG, CRP, complement (C1q and C3c), and dsDNA in glomerular basement membrane/subendothelial electron dense deposits in a small number of lupus nephritis patients. 

CRP has also been detected in kidneys undergoing acute rejection, and it was shown that CRP production could be induced in renal tubular epithelial cells [140]. Nakahara et al. [141] examined a wide variety of kidneys from children with various types of glomerular diseases. They found that CRP deposition was encountered more often in patients with proliferative diseases than in those with nonproliferative diseases. CRP deposition was primarily in the peritubular capillary walls and small vessels in the interstitium.

Recently evidence has been provided that CRP may play a pathologic role in certain mouse models of renal injury. Acute renal injury was induced in CRPtg mice and controls by clamping both renal pedicles for 30 min and then allowing reperfusion for 24 h. The transgenic mice had worse outcomes in all parameters measured [142]. The same research group had previously shown increased inflammation and fibrosis in CRPtg mice 3 days after induction of unilateral ureteral obstruction [143]. However, progression of renal injury at days 7 and 14 was equivalent for CRPtg and wild-type mice in this study.

SAP was shown to be a normal constituent of the glomerular basement membrane [144, 145]. SAP has been shown to potently inhibit renal fibrosis [146] *in vivo*. This effect was initiated by SAP binding to cell debris, followed by suppression of inflammatory macrophages through activation Fc*γ*RI and IL-10.

## 11. Pentraxins in Autoimmune Disease

A role for CRP in autoimmune diseases was suggested years ago when it was found that CRP was deposited in the nuclei of cells in the synovium of rheumatoid arthritis patients [147] and localized with polymorphonuclear cells in vasculitis [148] and experimental allergic encephalomyelitis [149]. These findings led to an exploration of the nature of the nuclear ligands recognized. Once it was established that CRP bound specifically to nuclear autoantigens including snRNPs, histones, and chromatin [28, 150–153], its role in SLE was investigated further. 

Our group initially hypothesized that CRP binding to nuclear autoantigens would promote their clearance and regulate the autoantibody response. We tested this in the (NZB x NZW) F1 female (NZB/W) mouse model of SLE [110]. NZB/W mice make a strong antichromatin and anti-DNA response and die of glomerulonephritis at about 9 months of age. In our study, NZB/W mice were injected with chromatin, which accelerates their autoimmune disease, in the presence or absence of CRP. The results showed a prolonged survival of mice injected with CRP and chromatin compared to chromatin alone, and a transient decrease in autoantibodies. However, the mice developed antibodies to CRP, which may have neutralized its later effectiveness. To circumvent this problem, Volanakis's group crossed a human CRPtg mouse strain [111] with NZB/W mice. They found that transgenic expression of CRP even at low levels (<5 *μ*g/mL) prolonged the survival of these mice by about 8 weeks. The CRPtg mice had decreased IgM anti-dsDNA, but increased IgG anti-dsDNA decreased renal disease. These results supported a protective role for CRP in SLE, but suggested that the mechanism probably was not suppression of autoantigen presentation or autoantibody responses.

Two additional papers showed that CRP given as a single injection of 200 *μ*g per mouse had a rapid and long-lasting protective effect on renal disease in both NZB/W and MRL/*lpr* mice [154, 155]. This work clearly established a predominant effect of CRP on ongoing renal pathology and showed a similar protective effect of CRP in accelerated nephrotoxic nephritis (NTN), an immune complex-mediated glomerulonephritis that is not autoimmune in nature [154]. The establishment of the NTN model allowed further analysis of the mechanism of CRP suppression of renal disease. The effect of CRP in NTN required macrophages, Fc*γ*RI, and IL-10, consistent with the induction of a regulatory macrophage phenotype [156]. In subsequent studies of experimental autoimmune thrombocytopenia, spleen cells or bone marrow macrophages treated with CRP *in vitro* transferred suppression of platelet clearance to recipient mice further supporting an Fc*γ*RI-dependent regulatory macrophage mechanism [157]. Further studies are needed to determine the steps subsequent to the induction of regulatory macrophages that result in long-term suppression of disease in SLE models. In the MRL/*lpr* mouse, mAb depletion experiments implicated regulatory T cells in the long-term suppression of renal disease [155].

Szalai's group examined the effect of both CRP deficiency (CRP^−/−^ mice) and overexpression (CRPtg) mice on the course of collagen-induced arthritis a model for human rheumatoid arthritis. CRP^−/−^ mice were more susceptible to induction of collagen-induced arthritis and developed more severe disease, whereas CRPtg mice were more resistant to disease induction and had a milder disease course [112]. These authors also showed a protective effect of CRP in the mouse experimental allergic encephalomyelitis model of human multiple sclerosis [158]. Similar to other inflammatory models, CRP increased the production IL-10. In addition CRP inhibited proliferation of encephalitogenic T cells and decreased production of inflammatory chemokines *in vitro*. They went on to demonstrate that CRP-mediated protection required the presence of the inhibitory Fc*γ*R, Fc*γ*RIIb [159]. The effect of transgenic rabbit CRP was also examined in mice generated by Jiang et al. [160]. Rabbit CRP was expressed under the PEPCK promoter, which is upregulated by diet manipulation. Induction of CRP expression led to a very marked inhibition of monoarticular antigen-induced arthritis. 

SAP has also been implicated in the pathogenesis of SLE largely because of studies that demonstrated that SAP bound to DNA and chromatin. It was postulated that SAP was responsible for clearance and degradation of these autoantigens from the blood [116]. Studies on SAP-deficient mice showed spontaneous antinuclear antibodies and severe glomerulonephritis, which supported this hypothesis [116]. However, this concept was challenged when an SAP “knock in” failed to correct the defect. It was determined that the background of the SAP^−/−^ mice was influenced by the process of gene knockout and that genes from the 129 strain contributed to the autoimmune manifestations [161]. SAP^−/−^ mice generated by a different group also spontaneously produced anti-nuclear antibodies but did not develop glomerulonephritis [126]. Recently, mouse SAP was reported to inhibit renal disease and autoantibody production in a model of SLE initiated by immunization of BALB/c mice with activated lymphocyte DNA in complete Freund's adjuvant [162]. Consistent with findings in CRP suppression of immune-mediated diseases, the mechanism of disease suppression by SAP involved the induction of regulatory macrophages producing IL-10 [163]. There is no indication of an involvement of SAP in SLE in man.

Another mechanism by which CRP is proposed to influence B cell activity is through shedding of membrane BLyS/BAFF by immune complex binding to Fc*γ* receptors. These investigators reported that Fc*γ* receptor cross-linking by either CRP or IgG IC induced the release of BLyS/BAFF from myeloid cells [164]. They further found that CRP, like IC, induced release of BLyS through the high affinity receptor for IgG, Fc*γ*RI.

CRP may display neoepitopes when bound to the surface of ELISA wells and some patients may develop antibodies that only react with these altered molecules. Bell et al. described autoantibodies directed towards CRP in patients who developed a type of illness resembling graft versus host disease following ingestion of contaminated cooking oil [165]. Surprisingly these antibodies reacted with cryptic epitopes of CRP but not to native CRP. Subsequently the presence of similar autoantibodies was reported in patients with SLE as well [166, 167]. The specificity for SLE is not complete as similar autoantibodies were seen in patients with chronic hepatitis C infection [168]. Although the clinical significance of these antibodies remains unknown, it has been proposed recently that complexes of CRP and anti-CRP along with anti-DNA antibodies may exacerbate inflammation by binding to necrotic remnants of apoptotic cells [169]. 

## 12. CRP in Cardiovascular Disease

Several years ago the identification of elevated baseline serum CRP as a predictor for cardiovascular events led to multiple studies by several groups to examine the role of CRP in mouse models of atherogenesis. Early studies suggested that CRP could facilitate the uptake of LDL by macrophages through opsonization. The interaction was reported to be dependent on micropinocytosis through Fc*γ*RIIa [26]. However, it remains controversial as to whether CRP binds to oxidized or otherwise modified LDL [25, 170, 171].

Human CRPtg or rabbit CRPtg mice were crossed onto mouse strains deficient in apolipoprotein E (apoE^−/−^) or low-density lipoprotein receptor (LDLR^−/−^) or CRP was infused into APOE*-Leiden mice. Although one study reported accelerated atherosclerosis in CRPtg/apoE^−/−^ mice [172], 5 subsequent studies found no effect [173–177]. A more recent study noted that the apoE^−/−^ mouse models have more severe hypercholesterolemia than humans as well as continuous low-grade inflammation and used a model of LDLR^−/−^ mice expressing apolipoprotein B100, crossed onto human CRPtg [178]. In this study the presence of human CRP slowed lesion progression and was thus atheroprotective. Recently CRP-deficient mice were developed by gene targeting. Studies done comparing CRP-deficient and -sufficient mice in ApoE^−/−^ and LDLR^−/−^ atherogenesis models produced results consistent with an atheroprotective role for CRP [113]. The combined findings of these studies indicate that CRP is either neutral or protective in atherosclerosis given the limitations of the mouse models.

Extensive epidemiological studies of human CRP polymorphisms do not support the hypothesis that genetically determined elevated baseline levels of CRP contribute to human cardiovascular disease [179]. However, this does not preclude participation of CRP-induced cellular responses within the atherosclerotic plaque or in reperfusion injury. 

## 13. Pentraxins and Monocytes and Macrophages

CRP and SAP bind preferentially to monocytes and neutrophils among human peripheral blood cells and opsonize targets for phagocytosis both directly through Fc*γ*R and Fc*α*RI [14, 96, 180, 181] and indirectly through the activation of complement [182]. Activation of peripheral blood mononuclear cells (PBMC) by CRP with production of inflammatory cytokines was originally reported by Ballou and Lozanski [183]. Subsequent studies identified a strong synergy between CRP and LPS as well as differential proinflammatory or anti-inflammatory cytokine release depending on whether PBMC or macrophages were used [184–186]. Interpretation of studies of pentraxins and cytokine induction is further complicated by the lack of receptor crosslinking by pentameric CRP and SAP. A recent analysis that addressed these issues identified IL-6, IL-10, and IL-8 release by monocytes activated by aggregated SAP [14]. These responses were inhibited by mAb to Fc*γ*RI and Fc*γ*RIII, and by Syk inhibitors. 

During infection macrophages may be exposed to both CRP and TLR ligands in the form of pathogen-associated or damage-associated molecular patterns (PAMPs or DAMPs). In this regard CRP acting through Fc*γ*RI and Fc*γ*RIIA enhanced PBMC production of proinflammatory cytokines, TNF-*α* and IL-1*β* in response to *S. pneumoniae *[91]. 

CRP has been injected into human volunteers to measure *in vivo* cytokine responses although the findings remain controversial. Bisoendial et al. injected healthy volunteers with 1.25 mg/kg recombinant human CRP. Cytokine profiles were generated by RT-PCR. He found upregulation of MMP9, MCP-1 (CCL2), uPA, MIP-1*α*, and I*κ*B*α* mRNAs in peripheral leukocytes [187]. However, these findings were disputed by Pepys who maintained that the injected CRP must contain contaminants [188]. It appears unlikely that uncomplexed CRP will induce proinflammatory events. However, CRP is frequently found at sites of tissue injury along with complement where it likely participates in the clearance of complexes and activation of cells through complement and Fc*γ*R. 

## 14. Pentraxins and Dendritic Cells

As CRP binds to pathogenic organisms and enhances their uptake by macrophages and dendritic cells (DCs), it was predicted to enhance antigen presentation and immunization. A model in which DCs pulsed with *S. pneumoniae* are used to immunize mice was used to test this hypothesis [189]. It was found that opsonization of *S. pneumoniae* with CRP prior to incubation with DC enhanced antibody responses compared to DC pulsed with unopsonized bacteria [190]. CRP had the greatest effect on the IgG secondary and memory responses to both protein (pneumococcal surface protein A) and polysaccharide (PC) antigens. CRP opsonization also increased the effectiveness of pulsed DC vaccination in protecting mice from intranasal challenge. The effects of CRP on *S. pneumoniae* uptake, antibody responses, and protection all required the FcR *γ*-chain.

CRP interactions with human DC have also been studied. A study by Zhang et al. [191] reported that CRP at low concentrations (10 *μ*g/mL) inhibited the differentiation of CD14^+^ monocytes into DC in the presence of GM-CSF and IL-4 as well as the maturation of immature DC by LPS. The inhibitory effect of CRP on DC differentiation was blocked by antibody to Fc*γ*RII. A second group [192] isolated myeloid DC from blood and showed that CRP at 10 *μ*g/mL or higher decreased the expression of the chemokine receptor CCR5 as well as the migration of these cells in response to the CCR5 ligand, MIP-1*β*. In contrast a third study [193] reported that CRP at very low concentrations (2 *μ*g/mL) induced further maturation of immature monocyte-derived DC, and that this also was inhibited by antibody to Fc*γ*RII. These conflicting results may in part be due to the use of commercial CRP preparations, which may contain denatured CRP as well as preservatives and contaminating LPS.

We have recently examined the effect of CRP on a distinct DC type, the plasmacytoid DC (pDC). Like myeloid DC, pDCs are found in low numbers in the blood. The pDCs play an important role in innate defense against viral infection by producing large quantities of type I interferon (IFN) [194]. More recently, pDCs have been implicated in the increased levels of IFN and the IFN-inducible gene expression pattern in the peripheral blood of patients with SLE [195]. In this case, autoantibody immune complexes containing nucleoprotein autoantigens induce IFN production. Immune complexes are taken up by pDC through Fc*γ*RIIa and activate intracellular TLR for RNA or DNA in the endosomal compartment to stimulate IFN synthesis. CRP binds to nucleoprotein autoantigens, snRNPs, and chromatin, as well as to Fc*γ*RIIa. However, we found that CRP-snRNP complexes did not induce IFN synthesis by pDC, and CRP inhibited the IFN response to autoantibody-snRNP complexes [196]. This inhibitory effect of CRP was associated with increased pDC maturation and with more rapid processing of IC into late endosome/lysosomes. IFN produced by pDC contributes to pathogenesis of SLE and other autoimmune diseases, so these results are consistent with the protective effect of CRP in mouse models of SLE [110, 139, 155, 156].

SAP binds to DNA, which is a TLR9 agonist. Recent studies showed that SAP binding to DNA blocks innate immune responses to DNA-based vaccines [197]. The authors found that, in mice tg for human SAP, T cell and antibody responses to DNA vaccines were decreased. The defective responses were shown to be the result of SAP binding to DNA, which facilitated uptake through Fc*γ*RI and Fc*γ*RIII. SAP prevented DNA binding to other DNA-binding molecules and inhibited activation of NF*κ*B and type I interferon responses in a human macrophage cell line.

## 15. CRP and Neutrophil Activation Chemotaxis and Phagocytosis

The interaction of CRP with neutrophils has been studied over many years. The first defined activity of CRP on neutrophils was its ability to opsonize both Gram-positive and Gram-negative pathogens [118, 198]. Mortensen et al. went on to show that CRP and complement acting in concert could induce phagocytosis of erythrocytes coated with the C-polysaccharide of *S. pneumoniae* (PnC) [182]. In 1985, Kilpatrick and Volanakis demonstrated that phagocytosis of PnC-coated RBC required activation of neutrophils by phorbol myristate acetate, a treatment that downregulates CD32A-dependent phagocytosis and increases Fc*α*RI-dependent binding and phagocytosis [181]. More recently, it has been shown that CRP-mediated phagocytosis by neutrophils may proceed through Fc*α*RI or Fc*γ*RIIA [96].

CRP has also been demonstrated to have inhibitory effects on neutrophils, particularly on neutrophil chemotaxis. For example, Webster's group found that CRP was able to inhibit neutrophil chemotaxis *in vitro* and *in vivo* [200, 201]. These effects were thought to be mediated through inhibition of p38 MAP kinase [202]. Zhong et al. also examined the effect of CRP on neutrophil chemotaxis with similar findings. He found that CRP inhibited neutrophil chemotaxis to IL-8 and fMLP (formyl-methionyl-leucyl-pheylalanine) chemotactic stimuli [203].

Zeller and Sullivan found that aggregated CRP could enhance chemoluminescence induced by IgG. This activity was strongly inhibited by antibodies to Fc*γ*RII/III [204, 205]. Unfortunately, many of these studies were performed before the characterization of the mouse and human Fc*γ*R and before blocking antibodies specific for each receptor were available.

More recently, the effects of CRP on phagocytosis have been reexamined with the finding that CRP also binds to CD89 [96]. Regulation of FcR on human neutrophils is rather complex and depends on several factors. Although human neutrophils normally express CD32A and GPI-anchored CD16B, they may express CD64 in response to signals like IFN-*γ*. It has also been reported that CD32A is maintained in a low affinity state unless stimulated by fMLP [199, 206]. In contrast CD89, the receptor for IgA, is upregulated by PMA resulting in preferential binding. CRP is capable of enhancing bacterial uptake through CD89 and upregulating its surface expression [96].

## 16. CRP Effects on the Vascular Endothelium

CRP has been extensively studied in cardiovascular disease and much of this work has revolved around its effect on the endothelium. One of the first studies suggested that CRP has a direct inflammatory effect on endothelial cells [207]. The investigators found that low concentrations of CRP in the presence of serum, acting through unknown mechanisms, would increase levels of adhesive molecules 10-fold. Unfortunately, the role of complement was not explored. These findings suggested that CRP might contribute to vascular injury and cardiovascular disease. 

A pathogenic role for CRP interaction was also supported by the finding that CRP could decrease eNOS expression in human aortic endothelial cells leading to attraction of monocytes to endothelial cells [208]. These activities were found to be due to CRP engagement of Fc*γ*R on the endothelial cells [209]. However, the finding of eNOS inhibition by CRP was challenged by others who suggested that CRP actually increased NO production *in vitro* and *in vivo* leading to a decreased response of phenylephrine-induced vasoconstriction [210]. CRP effects on the endothelium after experimental induced injury were also studied [211–213]. The prothrombotic effects of CRP in this model required Fc*γ*RI [214]. It was later shown by the same group that vascular damage induced by CRP required complement [215]. 

It has also been reported that CRP can induce apoptosis of vascular smooth muscle cells through stabilization of GADD153 mRNA [216]. These effects were seen at very low levels of CRP and it is unclear whether these *in vitro* findings are relevant *in vivo *although colocalization of CRP and GADD153 was found in atherosclerotic lesions.

Investigations of whether CRP contributes mechanistically to cardiovascular disease have been extensive and controversial. The absence of a clear effect in mouse models as described above has further impeded progress in this area. For a thorough discussion of the findings both supporting and disputing a role for CRP in atherogenesis, the reader is referred to three recent review articles [217–219].

## 17. CRP Genetics

The genes for the classical pentraxins lie on chromosome 1q23.2 in man. This is an immunological hot spot with genes for the Fc*γ*R lying close by at 1q23.3. This region is also associated with the risk for SLE in man and in mouse models of SLE. As discussed above, many studies have focused on the CRP gene due to its perceived involvement in cardiovascular disease. More than 100 SNPs in and around the CRP gene have now been identified. None of these polymorphisms is associated with the coding region of CRP and no variations in the protein sequence of CRP have been identified. However, polymorphisms in the noncoding regions in the promoter and the untranslated region have a substantial effect on baseline CRP levels. Polymorphisms in genes that stimulate CRP production like IL-6, IL-1, and several others also contribute to baseline CRP levels. Groups of CRP SNPs inherited together have been identified with five common major haplotypes in Northern European subjects. Two of these haplotypes are associated with high baseline CRP levels and two are associated with lower CRP levels [220]. Differences in acute phase levels of CRP are also influenced by these haplotypes [221]. Moreover CRP haplotypes have been linked to several disease states. In the case of cardiovascular disease, there is now at least some degree of consensus that genetically determined that baseline levels of CRP do not influence disease risk in a causative relationship despite their strong association reviewed in [222]. However, associations between CRP and individuals with genetically determined lower baseline levels of CRP are at increased risk of SLE and lupus nephritis [223, 224]. See the genetic association database at NCBI.

Since many of the effects of CRP in inflammatory states are related to Fc*γ*R and these receptors display polymorphisms association between these polymorphisms and disease risk efforts have been made to determine the importance of these Fc*γ*Rs in relation to CRP. Jönsen et al. studied Fc*γ*RIIA, Fc*γ*RIIIA, and CRP polymorphisms in relation to multiple SLE disease manifestations including glomerulonephritis [224]. They found associations between a low expressing CRP allele and more severe glomerulonephritis and an interactive effect between this CRP allele and the low IgG-binding Fc*γ*RIIIA allele (F/F).

## 18. Clinical Use of CRP Levels

CRP levels are used clinically in two different ways. The initial assays used to measure CRP in the circulation were relatively insensitive and for many years a positive value was used as an indication of inflammation or infection. CRP is an excellent marker of the acute inflammatory response and is used extensively for diagnosis and prognosis of rheumatologic and other diseases. CRP levels of 10 *μ*g/mL up to 500 *μ*g/mL can be seen in the acute phase response. CRP is routinely used to measure disease activity in rheumatoid arthritis and is part of the Disease Activity Score 28. Similarly measuring CRP levels is helpful in monitoring disease activity of various forms of vasculitis. However, CRP monitoring is of little value in measuring disease activity in SLE, scleroderma, polymyositis, or dermatomyositis where CRP levels do not correlate well with disease activity [225].

About 20 years ago highly sensitive assays were developed that could detect baseline CRP levels in apparently healthy individuals. These assays were said to measure high sensitivity (hs)-CRP although the only difference is in the ability to detect lower levels of CRP. There is little consistent evidence for the presence of CRP that is glycosylated or otherwise modified from native pentameric CRP in the circulation. The advent of the hs-CRP assay led to numerous studies that showed utility in individuals at risk for cardiovascular disease, metabolic syndrome, periodontal disease, and other chronic diseases associated with a low level of inflammation. The American Heart Association established ranges of CRP levels that were associated with risk of cardiovascular events [226]. These values probably reflect both genetic differences in CRP production and stimuli for its synthesis as well as underlying inflammation due to factors like periodontal disease and the metabolic syndrome. Whether there is a direct contribution of mildly elevated CRP levels to cardiovascular disease has been extensively debated [227]. Regardless of its role in pathogenesis it remains a strong marker of cardiovascular disease risk with equivalence to the widely measured risk factor, cholesterol [228].

## 19. CRP in Sepsis and Shock

As noted above CRP levels are highly elevated in patients with sepsis. The levels of CRP in sepsis have been shown to be related to mortality and organ failure [229]. In sepsis CRP was shown to participate in complement activation [230]. Recently it was reported that CRP strikingly downregulates the C5aR on PMN in patients with sepsis [231]. These finding are reminiscent of earlier findings that suggested that CRP could induce shedding of the IL-6 receptor on neutrophils [232] and suggest a regulatory role for CRP in the inflammatory response during sepsis.

These findings in human neutrophils are consistent with earlier findings in mouse models. An anti-inflammatory role for CRP was first shown in mouse models of lethal inflammation induced by LPS, platelet-activating factor (PAF), or TNF-*α* plus IL-1*β* [233]. These investigators developed a CRPtg strain of mice in which rabbit CRP was expressed under the diet-inducible phosphoenolpyruvate carboxykinase (PEPCK) promoter. They found that mice expressing acute phase levels of CRP were protected from lethal endotoxin shock as well as shock induced by PAF and TNF-*α* plus IL-1*β*. Subsequent studies indicated that CRP protection from PAF required an intact PC binding site and might be mediated by direct binding of CRP to the PC group on PAF [234]. 

Several other groups reported protection of mice from LPS shock by injection of human CRP as well [40, 121, 235]. These studies also showed that SAP, although it binds to LPS, was not protective [40]. CRP was protective and the mechanism required both activating and inhibitory Fc*γ*Rs [121]. This study demonstrated induction of a regulatory macrophage phenotype by CRP and LPS, similar to the regulatory macrophages induced by LPS and IC [236]. A similar anti-inflammatory pathway is induced by CRP in immune-mediated diseases as discussed above. 

Although these studies are consistent with a regulatory role for CRP in the acute phase response, it is more difficult to test this in humans. One study injected endotoxin into healthy volunteers with genetically different baseline CRP levels and measured the proinflammatory cytokines response (TNF-*α* and IL-6). Consistent with the results in the mouse models, individuals with higher CRP levels had lower TNF-*α* and IL-6 responses to LPS injection [237]. 

Traumatic injury induces dramatic changes in both pro-inflammatory mediators that can result in shock as well as anti-inflammatory mediators that can suppress the immune system [238]. Monocytes and macrophages are key initiators and regulators of innate immune responses following trauma. In a study of 50 trauma patients, we observed an increase in an activated monocyte/macrophage population (CD14^high^CD16^+^CD163^+^) in the blood that was highly correlated with CRP levels, as well as M-CSF and TGF-*β* [239], M-CSF and TGF-*β* found in trauma plasma could induce this phenotype in normal monocytes. Although it was not essential for inducing the phenotype, CRP activated M-CSF differentiated monocytes to produce anti-inflammatory cytokines, IL-10 and IL-1RA. These findings are consistent with a role for CRP in the anti-inflammatory response following trauma that helps prevent shock.

## 20. SAP in Disease

The other major member of the pentraxin family is SAP. In this section I focus on properties of SAP related to human disease. Unlike CRP, SAP is a constitutively expressed protein in man that is normally present at about 40 *μ*g/mL in blood. SAP was named for its physical association with amyloid deposits associated with various forms of amyloidosis, which is associated with a variety of inflammatory hereditary, malignant and infectious conditions. These amyloid deposits are normally detected by biopsy of the affected organ and fluorescent staining. Amyloid deposits progressively affect organ function by massively infiltrating the parenchyma.

The function of SAP in the amyloid plaque is not completely understood. It has been proposed that SAP serves to stabilize the amyloid fibrils against degradative enzymes [240]. Like CRP, SAP is very resistant to enzymatic attack due to its tightly packed structure. In mice targeted deletion of the SAP gene leads to delayed amyloidogenesis in a reactive model of systemic amyloidosis [115]. This finding has led to several approaches designed to block this stabilization by depleting SAP systemically. It has been shown that the extent and localization of amyloid deposits may be determined by imaging studies that use injected, labeled SAP as a marker [43]. Treatment of amyloidosis with agents that clear SAP from circulation along with anti-SAP antibodies has been tried and found to be effective in an animal model [241]. More recently, similar studies were done with patients suffering from amyloidosis [242]. However, the results of follow-up studies are unavailable so far. Similar studies have been reported in Alzheimer's disease [243]. However, the clinical utility of this agent remains unknown.

There is a growing body of studies suggesting unique properties of SAP in wound healing and regulation of fibrosis. Pilling et al. first demonstrated that SAP could prevent the generation of fibrocytes *in vitro* and suggested that this might contribute to delayed wound healing and facilitated connective tissue disease [244]. They went on to show that this effect was mediated by binding Fc*γ*R and specifically to Fc*γ*RI [245]. These studies have led to a series of human clinical studies related to treatment of chronic fibrosing conditions such as interstitial pulmonary fibrosis, macular degeneration and myelofibrosis. These are severe irreversible conditions, and any new therapeutic agents would be welcomed. An *in vivo* correlate of this function was shown as well. SAP was found to downregulate the conversion of monocytes to fibroblasts in a mouse model of fibrotic cardiomyopathy [246].

## 21. Summary

The pentraxins are a family of evolutionarily conserved proteins that trace their evolutionary roots back to the early invertebrates. They have evolved along with the innate and adaptive immune system interacting with the ancient complement system and the Fc receptors. Their best described role is in host defense although they are important pattern recognition molecules for altered self-antigens as well. 

**Figure 4:**
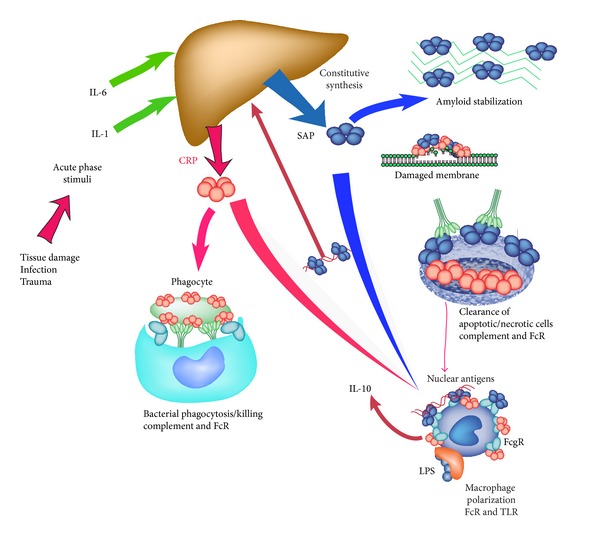
Overview of the major activities of the pentraxins.  Both CRP and SAP are predominantly serum proteins, produced in the liver. In man SAP is constitutive and CRP is a major acute phase reactant. Both contribute to host defense as direct opsonins and through complement activation. Both bind to ligands exposed during cell death and tissue damage leading to opsonization and removal. In addition to these activities many studies support a role for pentraxins in regulating the inflammatory response to immune complexes and TLR agonists. This regulation is initiated by pentraxin interactions with Fc*γ*R and mediated by polarized macrophages.

The pentraxins have been studied for over 80 years now and we have learned a great deal about their structure, function and evolution. Despite this intensive study we have only recently begun to understand their role in disease and host defense (Figure 4). Figure 4 provides a cartoon representation of the major known properties of the pentraxins. CRP production is stimulated inflammatory events whereas SAP is constitutively expressed. Both pentraxins activate complement and play a role in host defense although this is much better studied for CRP. Both CRP and SAP also bind to damaged cells and nuclear components and facilitate their safe removal in a nonimmunogenic manner. When binding of pentraxins to multivalent ligands leads to FcR crosslinking, macrophage polarization is seen, which in the case of CRP suppresses inflammation and in the case of SAP prevents fibrosis. Overall the pentraxins provide a regulatory pathway to control the inflammatory response to tissue injury.

## Figures and Tables

**Table 1:** Comparison of the properties of the pentraxins: C-reactive protein (CRP) and serum amyloid P (SAP).

	C-reactive protein (CRP)	Serum amyloid P (SAP)
Fc receptor binding	Yes	Yes
Calcium-dependent ligand binding	Yes	Yes
Complement activation through C1q	Yes	Yes
Ligands	Phosphocholine snRNP (Sm, RNP) Histones Apoptotic cells Oxidized LDL	Phosphoethanolamine DNA, chromatin Heparin Apoptotic cells Amyloid fibrils
Major synthetic site	Liver	Liver
Inducers	IL-6 (acute phase reactant)	Constitutive
Structure	Cyclic pentamer 115,135 Da Each subunit 23,027 Da 206 amino acids	Cyclic pentamer 127,310 Da Each subunit 25,462 Da 204 amino acids
Glycosylation	No	Yes
Chromosomal location	1q23.2	1q23.2

**Table 2:** Overview of pentraxin receptors.

Receptor	Cells		Other ligands	Functions
Fc*γ*RI (CD64)	Monocytes, macrophages, DC, inducible on PMN	Activating	High affinity for IgG	Antibody-dependent cell-mediated cytotoxicity Phagocytosis

Fc*γ*RIIA/C (CD32A/C)	Monocytes, macrophages, DC neutrophils, platelets	Activating	IgG	Antibody-dependent cell-mediated cytotoxicity Phagocytosis Platelet activation

Fc*γ*RIIB (CD32B)	B lymphocytes, macrophages, DC	Inhibitory	IgG	Regulation of responses through immunoreceptors

Fc*γ*RIIIA (CD16A)	Macrophages, some monocytes, NK cells	Activating	IgG	Antibody-dependent cell-mediated cytotoxicity Phagocytosis

Fc*γ*RIIIB (CD16B)	PMNs	GPI-linked	IgG	Immune complex binding Activation of Fc*γ*RIIA

Fc*α*RI (CD89)	Monocytes, macrophages, DC neutrophils, platelets	Activating/inhibitory	IgA	Phagocytosis Regulation of responses through other receptors

## Abbreviations

CRP: C-reactive protein
DC: Dendritic cells
Fc*γ*R: Fc*γ* receptors
fH: Factor H
Fc*α*RI (CD89): The IgA receptor
ITAM: Immunoreceptor tyrosine-based activation motif
LPS: Lipopolysaccharide
PC: Phosphocholine
NPTXI and NPTXII: Pentraxin I and II
PTX3: Long pentraxin 3
snRNPs: Small nuclear ribonucleoproteins
SAP: Serum amyloid P component
SPR: Surface plasmon resonance
SLE: Systemic lupus erythematosus
TLR: Toll-like receptor
tg: Transgenic
NZB/W (NZB x NZW): F1 female mouse model of SLE
apoE: Apolipoprotein E
LDLR: Low density lipoprotein receptor
PBMC: Peripheral blood mononuclear cells
pDC: Plasmacytoid DC
PEPCK: Phosphoenolpyruvate carboxykinase promoter.
